# Harmonic quantum cascade laser terahertz frequency combs enabled by multilayer graphene top-cavity scatters

**DOI:** 10.1515/nanoph-2023-0912

**Published:** 2024-02-29

**Authors:** Manuel Alejandro Justo Guerrero, Omer Arif, Lucia Sorba, Miriam Serena Vitiello

**Affiliations:** NEST, CNR – Istituto Nanoscienze and Scuola Normale Superiore, Piazza San Silvestro 12, 56127, Pisa, Italy

**Keywords:** terahertz, quantum cascade lasers, harmonic frequency combs, quantum optics

## Abstract

Optical frequency comb synthesizers, operating in the harmonic regime, are metrological sources in which the emitted optical power is concentrated in a few modes, spaced by several multiples of the cavity free spectral range (FSR). This behavior reflects in a large correlation degree and, in principle, in an increased optical power per mode. In miniaturized quantum cascade lasers (QCLs), harmonic frequency combs (HFCs) are hence particularly attracting to explore quantum correlation effects between adjacent harmonic modes, enabled by the inherently large gain media third-order *χ* ^(3)^ Kerr nonlinearity, even if controlled generation of stable HFCs of predefined order, is typically demanding in such electrically pumped sources. Here, we demonstrate stable 2^nd^ order and 3^rd^ order HFC emission in terahertz frequency QCLs by respectively patterning an individual or a couple of equally spaced distributed multilayer graphene absorbers on the top metallic waveguides.

## 1 Introduction

In the rapidly advancing landscape of quantum science and technology, the development of quantum devices operating at terahertz (THz) frequencies has emerged as a frontier that prospects an immense potential for a variety of applications, including communications and imaging [1], sensing [2], materials science, and metrology [3], [4], [5], [6]. Among the most recent developments of THz technologies, the experimental discovery that THz frequency quantum cascade lasers (QCLs), miniaturized quantum sources, can emit optical frequency combs spontaneously [7] has opened new areas for fundamental study and application-oriented developments [8]. THz frequency combs (FCs), characterized by a series of evenly spaced and coherent spectral lines, may indeed lead to a paradigm shift in the spontaneous generation of quantum states of light [9], providing a fundamental metrological tool to generate in phase optical modes with tailored frequencies of pre-defined spectral spacing.

Together with the standard operation on their fundamental order, where the mode spacing matches the cavity round trip frequency, QCL can also operate as harmonic frequency combs (HFCs). In such a state, the emitted power is concentrated in a few modes separated by a multiple of the laser’s free spectral range [10], [11]. In this latter case, the huge (∼10−15 m^2^ W^−1^) [12] third-order Kerr nonlinearity promotes the coherent coupling between the amplitude and phase of light, leading to both fundamental FC and HFC, mode proliferation. So far, it has been observed in both mid-infrared [13], [14], [15] and THz QCLs [9], [16], [17], [18], that those states (fundamental and HFC) can co-exist in the same device, alternating between them with irregular mode locking, depending on the driving current and operating temperature [16]. Therefore, reaching a pure and stable HFC regime in electrically driven QCLs, across almost their entire dynamic range, has been challenging so far. In the mid-infrared, the controlled generation of the harmonic states of a specific order in QCLs has been accomplished through optical seeding [10] or by engineering localized defects in a ring laser cavity [19]. More recently, we have demonstrated effective control of the HFC emission in a THz Fabry–Perot (FP) QCL by implementing regularly spaced defects, comprising a thin lossy metal, along the laser top contact, distributed via an accurate tailoring, along the laser bar [20]. Here, we demonstrate HFCs of pre-defined order in FP QCLs based on a homogeneous active region diagonal laser design and emitting at THz frequencies. The device architecture comprises localized multilayer graphene absorbers, equally distributed along the laser cavity, whose number *n* fixes the expected harmonic order (*n* + 1) of the devised HFC. The optical non-linear effects of graphene at THz frequencies enhance the frequency-locking and phase-locking of the harmonic modes [21], [22] affecting the spatial dependence of the intermode beat note intensity along the cavity, originating from the interference of any combination of lasing frequency comb modes. Furthermore, graphene simultaneously acts as an efficient saturable absorber [23], [24]. Stable and controlled 2^nd^ and 3^rd^ order harmonic operation between 45 % and 60 % of the QCL operational range is demonstrated, with an optical power per mode >0.39 mW, around a central frequency of 3 THz. The ability to control the harmonic frequency comb generation in THz QCLs, by design, opens the door to novel applications such as the generation of multi-mode compressed states of light. This represents a key route for the widespread use of THz radiation in unexplored domains and for metrological, spectroscopic, and quantum imaging applications.

## 2 Results and discussion

**Figure 1:**
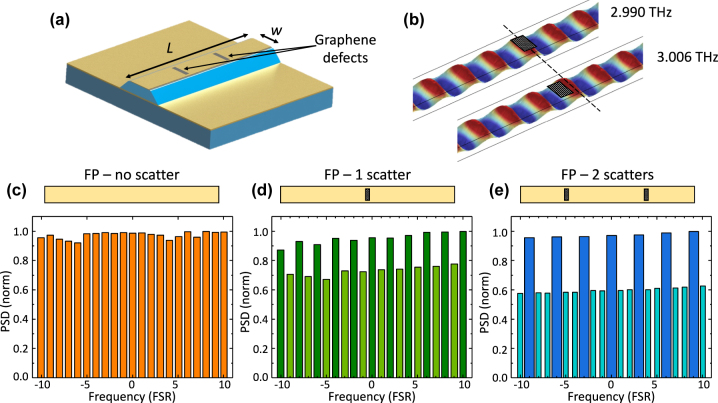
Device architecture and simulations. (a) Three-dimensional schematic of the device architecture. The top contact of the metal-metal QCL is patterned with a periodic array of *n* rectangular slits covered with multiple (3) graphene layers. To obtain the *n* ^th^ harmonic response, the slits are spaced by a distance *L*/(*n* + 1). (b) Schematic representation of the electric field distribution inside the cavity with one graphene scatter on the top contact. The dashed line points out the relative alignment of the field peak and the scatter at two different frequencies. (c–e). Power spectral density of the field simulated in different cases: the pristine cavity without any aperture on the top metal contact has a PSD evenly distributed across all of the modes (c); when the scatters are introduced (d and e), the modes whose field peaks are not aligned with the scatters exhibit a notable reduction of the intensity, resulting in a harmonic modulated spectrum. The spectra are normalized by the free spectral range (FSR) of the cavity and centered around 3 THz.

To understand the effect induced by the graphene scatters on the generated TM_00_ QCL modes, we perform three-dimensional finite element method simulations (Comsol Multiphysics). Further details on the simulation parameters are provided in the Supplementary Material. Figure 1(a) shows the schematic model of an edge emitting QCL of 2.5 mm length (L), 70 µm width (w), and 17 µm active region thickness (t), having two Ni side stripes and two graphene-coated scatters on the QCL top contact. As a function of the number of scatters (*n*), the laser bar is divided into *n* + 1 sections of the same length. The simulation results show that the optical modes, sustained by the resonance cavity, are effectively perturbed by the presence of the graphene scatters. These scatters induce additional losses and internal reflections in the cavity; consequently, the intensity of the modes supported by the cavity is modulated. Figure 1(b) shows the spatial electric field (*E*_*z*_) distribution inside the cavity of a QCL with one graphene scatter located at the center of the laser bar (*L*/(*n* + 1)). The field distribution of two contiguous frequency modes located at 2.990 THz and 3.006 THz, is shown. The graphene scatter on the top contact was highlighted in Figure 1(b) for the purpose of showing the shifting of the field phase with respect to the scatter, as indicated by the dashed line. At 2.990 THz, the scatter position matches the electric field peak, whereas at 3.006 THz the graphene scatter mismatches with the peak of the electric field. Henceforth, these two scenarios defined what we indicated as aligned and misaligned cases, respectively. The simulations reveal that the modulation of the modes supported by the cavity is highly dependent on the position of the scatter and on the phase of the electric field, resulting in a larger intensity modulation when the phase of the electric field is misaligned with respect to the graphene scatter position. Furthermore, the modulation of the modes is also dependent on the number of graphene scatters present on the top contact [20]. Figure 1(c–e) shows the field power spectral density (PSD) at the center of the QCL bandwidth [25], [26]. Figure 1(c) depicts the PSD calculated in the reference structure, without any scatter on the top metallic contact, in a range of 20 free spectral range (FSR). In Figure 1(d) and (e) we show the calculated PSD for a cavity with 1 and 2 equally spaced graphene scatters, respectively. The PSD from the simulated reference cavity reveals a series of modes of comparable intensity, equidistantly spaced every 1 FSR. Besides, the PSD from the cavities with 1 and 2 top graphene scatters, exhibit a series of modes visibly modulated as a function of *n*, corresponding, as expected, to the second and third harmonics of the QCL, respectively. However, compared to the case of lossy metal defects [20], the graphene scatters induce a more stable attenuation of the PSD of the non-harmonic modes, leading to a significantly enhanced suppression ratio of the non-harmonic modes. Moreover, the analysis of the electric field distribution inside the FP cavity unveils that the presence of the graphene scatters induces a redistribution of the field components of the sustained modes, reducing the average *E*_*z*_ field amplitude and increasing the *E*_*x*_ and *E*_*y*_ amplitudes. Being the photons interacting with the inter-subband laser gain the transverse magnetic polarized ones [19], only the electric field parallel to the growth direction (*z*) is responsible for laser action.

In order to determine the field redistribution, we evaluate the ratio *E*_*z*_/*E*_tot_ inside the cavity volume, where *E*_tot_ represents the sum of the three field components (see Supplementary Material). This analysis shows that, compared to the scatters-free cavity, *E*_*z*_/*E*_tot_ decrease by only 9 % and 4 % for the harmonic modes (aligned case) in a cavity with one and two graphene scatters, respectively. In contrast, *E*_*z*_/*E*_tot_ drops by 24 % and 11 % for the non-harmonic modes (misaligned case). Finally, we calculate the radiative losses in the cavities from the three dimensional (3D) quality factor (see Supplementary Material). The radiative losses follow a similar trend as the *E*_*z*_ */E*_tot_ ratio, being larger, on average, for the non-harmonic modes rather than for the harmonic ones, either for the cavity with one or two graphene scatters. Nevertheless, the calculated radiative losses show a reduction of up to 24 % if compared to the losses calculated when the same FP laser cavity is patterned with nickel defects, meaning that a larger intracavity power is expected in the present configuration. If we define as harmonic modulation the ratio of the average radiative losses in harmonic and non-harmonic modes, we get that for the case of a cavity with one patterned graphene scatter the modulation is about 27 %, whereas it is around 5 % in the cavity with two graphene scatters (see Supplementary Information).

A set of QCLs with a patterned number of graphene scatters in the *n* = 0–2 range was fabricated in a double metal FP cavity architecture. The employed active region consists of a 10 µm thick GaAs/AlGaAs heterostructure grown by molecular beam epitaxy (MBE) on a semi-insulating GaAs substrate. The diagonal active region is based on a longitudinal optical phonon-assisted interminiband transition with a gain bandwidth cantered at 3 THz. Devices were fabricated with metal-metal waveguides starting with an Au–Au thermocompression wafer bonding of the MBE active region onto a highly doped GaAs substrate. The laser bars were then defined via a combination of optical lithography and metal deposition. The Cr/Au (10 nm/150 nm) top metal contact is defined with two adjacent lateral stripes patterned 5 µm away the two side of the ridge and covered with a 5 nm thick Ni layer that defines lossy side absorbers, useful for suppressing high-order lateral modes by increasing their threshold gain [27].

**Figure 2:**
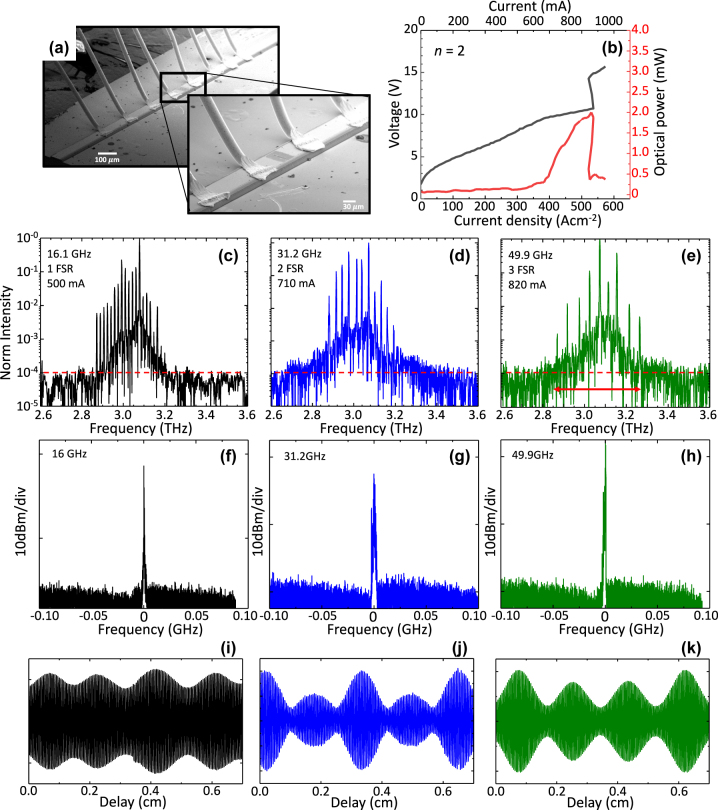
Optical chatacterization and emission spectra. (a) Scanning electron microscope (SEM) images of a double metal QCL with one graphene scatter on the top contact. (b) Voltage-current density and light-current density characteristics of the device with two graphene scatters measured at a heat sink temperature of 17 K. The optical power was measured using a broad-area THz absolute power meter (TK Instruments, aperture 55×40 mm^2^. (c–e) Fourier transform infrared (FTIR) emission spectra of the reference device without any top surface patterned scatters. (c), and of the QCL with 1 and 2 top surface graphene scatters (d and e). The red dashed lines mark the noise level. The spectra become perfectly harmonic, according to the distance between the graphene scatters (FSR = *n* + 1). All spectra are acquired under vacuum in continuous wave at a heat sink temperature of 20 K, and while driving the QCL at the current mentioned in the panel (c–e). (f–h) Intermode beat note of the reference laser (f) and of the device with *n* = 1 (g) and *n* = 2 (h), collected with an RF spectrum analyzer, under the same condition of the corresponding spectra. (i–j) Corresponding interferograms (IFG) of the spectra in panel c (i), d (j) and e (k).

The Cr/Au (10 nm/150 nm) top metal contact comprises equally spaced rectangular apertures having a dimension of 3 µm × 42 µm that are finally covered by transferring three layers of graphene (Graphenea), grown by chemical vapor deposition, having a ≈(3 × 0.35) nm thickness. The 60–70 µm wide and 2.5 mm long double metal FP laser cavities were then indium soldered on copper plates and wire bonded. Scanning electron microscope (SEM) images of a prototypical device are shown in Figure 2(a). The devised QCL HFCs were then mounted onto the cold finger of a He flow cryostat.

The voltage-current density (VJ) and light-current density (LJ) characteristics were measured in continuous-wave at a fixed heat sink temperature of 21 K. The fabricated devices show an average threshold current density *J*_th_ = 352 ± 4.4 mA/cm^−2^ which slightly increases with *n*, with a slope efficiency of 26.58 ± 2.13 mW/A (see Figure 2(b) for *n* = 1) and a maximum wall plug efficiency of ≈0.067 %, respectively (see Figure 2(b) for *n* = 1). Slope efficiencies are extracted from the light-current characteristics acquired by placing a pyroelectric detector having a 3 × 3 mm^2^ area, in front of window of the cryostat, at about 2 cm distance from the laser facet. The divergent double-metal far field profile is hence used to normalize the signal measured by the lock-in which is converted to an optical signal after calibrating the power measurement. The power scale refers to the power extracted from one facet and is normalized considering the 75 % transmission of the cryostat window. A table summarizing all device performances is reported in the Supplementary Material.

The VJ and LJ curves of a reference QCL fabricated with an identical architecture but with *n* = 0 are presented in the Supporting Material. The optical power delivered by the lasers is about 2.39 ± 0.6 mW, with a dynamic range *J*_max_ */J*_th_ = 2.31 ± 0.3, where *J*_max_ represents the highest current density at which the QCL exhibits lasing (see Supplementary Material). A broad-area THz absolute power meter (TK Instruments, aperture 55 × 40 mm^2^) was used to measure the peak optical power.

Fourier transform infrared (FTIR) spectra where recorded under-vacuum (Bruker Vertex v80), with a resolution of 2.25 GHz. The FTIR spectra of the different devices are shown in Figure 2(c–e), as well as their corresponding acquired interferograms in Figure 2(f-h). The spectrum of the reference (*n* = 0) QCL exhibits frequency comb behavior, with a set of 23 optical modes uniformly separated by 16.1 GHz (1 FSR), covering an optical bandwidth of 0.4 THz. The spectra of the lasers with *n* = 1 and *n* = 2 (Figure 2(d) and (e)), on the other hand, reveal a pure harmonic response with an optical mode repetition that matches the n^th^ harmonic order, predicted by simulations: 2^nd^ harmonic for *n* = 1 and 3^rd^ harmonic for *n* = 2. Additionally, from the interferograms it can be observed a peculiar and symmetric pattern that changes with *n*. In the reference device, the interferogram exhibit an almost regular pattern, while the interferogram from the 2^nd^ order HFC shows a pattern composed by two lobes of different intensities which alternate periodically and symmetrically, and the interferogram of the 3rd order HFC shows a pattern composed by three lobes repeated periodically and symmetrically, two of the lobes are of the same intensity and the third one is of larger intensity. The behavior retrieved in the case of *n* = 2 is regularly found in the case of emission of pulses [28], a condition that, in the present devices is prone to happen due to the presence of the distributed graphene saturable absorbers. It is worth mentioning that, in the case of HFC with localized Ni defects [20] the retrieved interferograms appear, conversely, clearly symmetric, independently of the harmonic order, as expected in the case of purely frequency comb states [16] without any signature of a periodic wave package that could be indicative of generated pulses. The corresponding beat notes measured for each HFC are shown in Figure 2(f–h).

**Figure 3:**
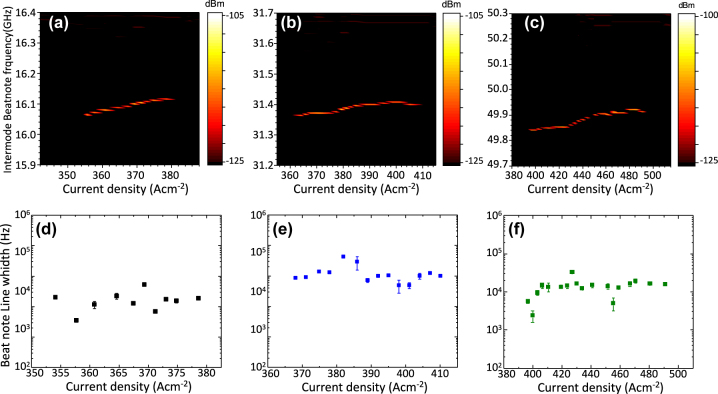
Intermode beatnote. (a–c) Intermode beat note maps as a function of the driving current, measured at 20 K for the FC QCL fabricated with any defect (a), with one graphene scatter (b), and two graphene scatters (c). (d–f) Beat note linewidths as a function of the continuos wave driving current, measured at 20 K for the FC QCL fabricated with zero (d), one (e), and two (f) graphene scatters, respectively.

To determine the harmonic frequency comb regime of the fabricated devices, we traced the intermode beat note frequency as a function of the continuous-wave driving current. The devices were operated at 20 K and the RF signal was detected using a bias tee (Tektronix AM60434) connected between the QCL and a spectrum analyzer (Rohde and Schwarz FSW43). To extend the operation range of the spectrum analyzer up to 60 GHz, it was equipped with a mixer (Rohde and Schwarz FS-Z60). The intermode beat note maps of the reference (*n* = 0) and the two lasers with *n* = 1 and *n* = 2 are shown in Figure 3(a–c). In the case of *n* = 0 cavity, it is observed a single BN around 16.1 GHz (Figure 3(a)) with ≈19 dBm average intensity and a narrow linewidth (LW) ≈10 kHz, which persists for 27 A cm^−2^ (from 354 to 381 A cm^−2^). This range corresponds to the 25 % of the operational range. For the HFC with one defect, a narrow (≈13 kHz) BN appears around 31.2 GHz (2FSR) with a signal to noise ratio of ≈18 dBm, persisting for 48 A cm^−2^, from 362 to 410 A cm^−2^ which corresponds to the 48 % of the operational range (3b).

Finally, the HFC with *n* = 2 shows a ≈49.9 GHz (3 FSR) mode spacing and reveals a consistent and narrow (≈10 kHz) BN (≈21 dBm) that persists across 94 A cm^−2^, from 396 to 490 A cm^−2^ corresponding to >60 % of the device operational range (Figure 3(c)). The retrieved increase of the HFC operational range as a function of *n*, is very likely an effect of the Fresnel reflection at the graphene scatters. Being this reflection spatially separated from the saturable absorption occurring inside graphene, it might contribute to frequency comb stabilization in ranges dominated by dispersion, through the same mechanism as the fast saturable gain in the QCL active region.

All of the beat note maps were acquired using a resolution bandwidth RBW = 300 Hz, video bandwidth VBW = 500 Hz, and sweep time SWT = 60 ms. Apart from the reported harmonic BNs, no additional beatings were detected across the operational current ranges studied for each device. This was determined by analyzing the RF spectra at subharmonic frequencies along the operational range of the devices. This confirms that the investigated QCLs with graphene scatters operate as stable self-starting HFC without implementing any dispersion compensation mechanism [29], [30], [31], [32], or any strategy to modulate the intra-cavity field [33]. Moreover, the HFC regime is expanded as the number of multilayer graphene scatters increases, resulting in a broader spectral coverage that varies from 0.28 THz (*n* = 0 QCL) to 0.4 THz (*n* = 2 QCL).

## 3 Conclusions

In conclusion, by implementing a pre-defined number of multilayer graphene scatters on the top contact of THz QCLs, we introduce a spatially periodic modulation of the optical gain that promotes the emission of harmonic modes. Pure HFCs at the 2^nd^ and 3^rd^ order are demonstrated up to >60 % of the device operational current range. The 2^nd^ harmonic frequency combs show 11 optically active modes evenly spaced by 31.2 GHz (2 FSR), delivering ≈270 µW of optical power per comb tooth. On the other hand, the 3^rd^ harmonic frequency combs develop eight optical modes active, separated by 49.9 GHz (3 FSR), delivering ≈390 µW per comb tooth, a remarkable high value for metrological and spectroscopic applications. Importantly, a central multilayer graphene scatterer, which also acts as an efficient THz saturable absorber, induce a visible change in the continuous-wave emission interferogram, with signature of periodic and regular wave packages, indicative of possible self-starting emission of pulses in the second order HFC.

## Supplementary Material

Supplementary Material Details
