# Supplementary material for: Harmonic quantum cascade laser terahertz frequency combs enabled by multilayer graphene top-cavity scatters
Source: Nanophotonics. 2024 Feb 29;13(10):1835–41. doi: 10.1515/nanoph-2023-0912 (PMC11501973; doi:10.1515/nanoph-2023-0912)
Supplement: Supplementary file 1 — Supplementary Material Details [file j_nanoph-2023-0912_suppl_001.docx]

**Supplementary Material**

**Harmonic quantum cascade laser terahertz frequency combs enabled by multilayer graphene top-cavity scatters**

M. Alejandro Justo Guerrero^1^, Omer Arif^1^, Lucia Sorba^1^ and Miriam Serena Vitiello^1^*

*^1^ NEST, CNR - Istituto Nanoscienze and Scuola Normale Superiore, Piazza San Silvestro 12, 56127, Pisa, Italy*

1. **Finite elements simulations**

The frequency domain finite element method simulations are performed using a commercial software (Comsol Multiphysics 6.0), employing the wave optics module in eigenfrequency and frequency domain modes. The double metal-metal Fabry Perot cavity is simulated in three dimensions, assuming a 2.5 mm cavity length, 70 μm in width, and 17 μm in thickness. The cavity is enclosed in a larger volume of the same length, with 210 μm in width and 51 μm thickness. Furthermore, two volumes are placed at both ends of the cavity to allow the electric field to freely propagate away from the cavity facets. Finally, 3 μm × 42 μm rectangular slits and the 3μm wide nickel side absorbers are drawn on the top surface of the cavity.

The refractive index of the active region is set at n_AR_ = 3.6. The real and imaginary parts of the nickel side absorber refractive index are set to *n*_Ni_ = 30 and *k*_Ni_ = 50, respectively. The graphene on the top contact defects was defined by its scattering complex refractive index, *n*_Gr_($\nu$), taken from reference [1S]. The cavity volume surroundings are defined by a refractive index *n*_Air_ = 1. To simulate the gold interface, the top and bottom boundaries of the cavity have been defined as perfect electric conductors (PEC). The boundaries of the nickel side absorbers and graphene scatters are defined independently utilizing the transition boundary condition and a layer thickness of 5 nm. To simulate an infinitely large surrounding, all external boundaries are considered as scattering boundaries. The boundaries including the facets of the Fabry Perot cavity are defined as ports in frequency domain simulations, one as an input port where the TM_00_ mode is injected into the cavity and the other as a sensing port. Finally, the mesh properties were optimized to obtain a good resolution in the simulation frequency range of 2.5 to 3.5 THz.

Figure S1 shows the spatial distribution of the electric field in the QCL cavity, evaluated from the simulations, at 2.990 THz and 3.006 THz, which correspond to adjacent modes in which the *E_z_* field is aligned and misaligned with the slits, respectively. On the left side of figure S1 (a,b), the magnitude of the electric field distributed along the cavity is depicted from the top view (x-y) and cross-sections (y-z and x-z) in two different conditions: when the defects favour the existence of the third harmonic in the cavity (top) and when they do not (bottom). The two graphene defects on the cavity top surface are indicated by the white dashed rectangles. When the maximum of the electric field is aligned with the defects, the condition that favours the sustaining of modes at the third harmonic arises. When defects are misaligned with the maximum of the electric field, sustaining the modes that do not match the third harmonic frequency becomes more difficult, due to the alterations of the electric field distribution. The central panel of figure S1(c) illustrates how the existence of defects causes the power spectral density (PSD) to be greatly reduced for the modes that are not at the third harmonic. The amplitude of the *E_z_* field distribution at 2.990 and 3.006 THz, which has been amplified by a factor of 2, is shown on the right side of figure S1 (d). When one graphene scatter on the top contact is aligned with a minimum (blue region) of the *E_z_* field, the effect of the scatters on the field is seen in the panel on top. The reverse scenario, where the scatters and the *E_z_* field peaks are misaligned, is depicted in the bottom panel.

**
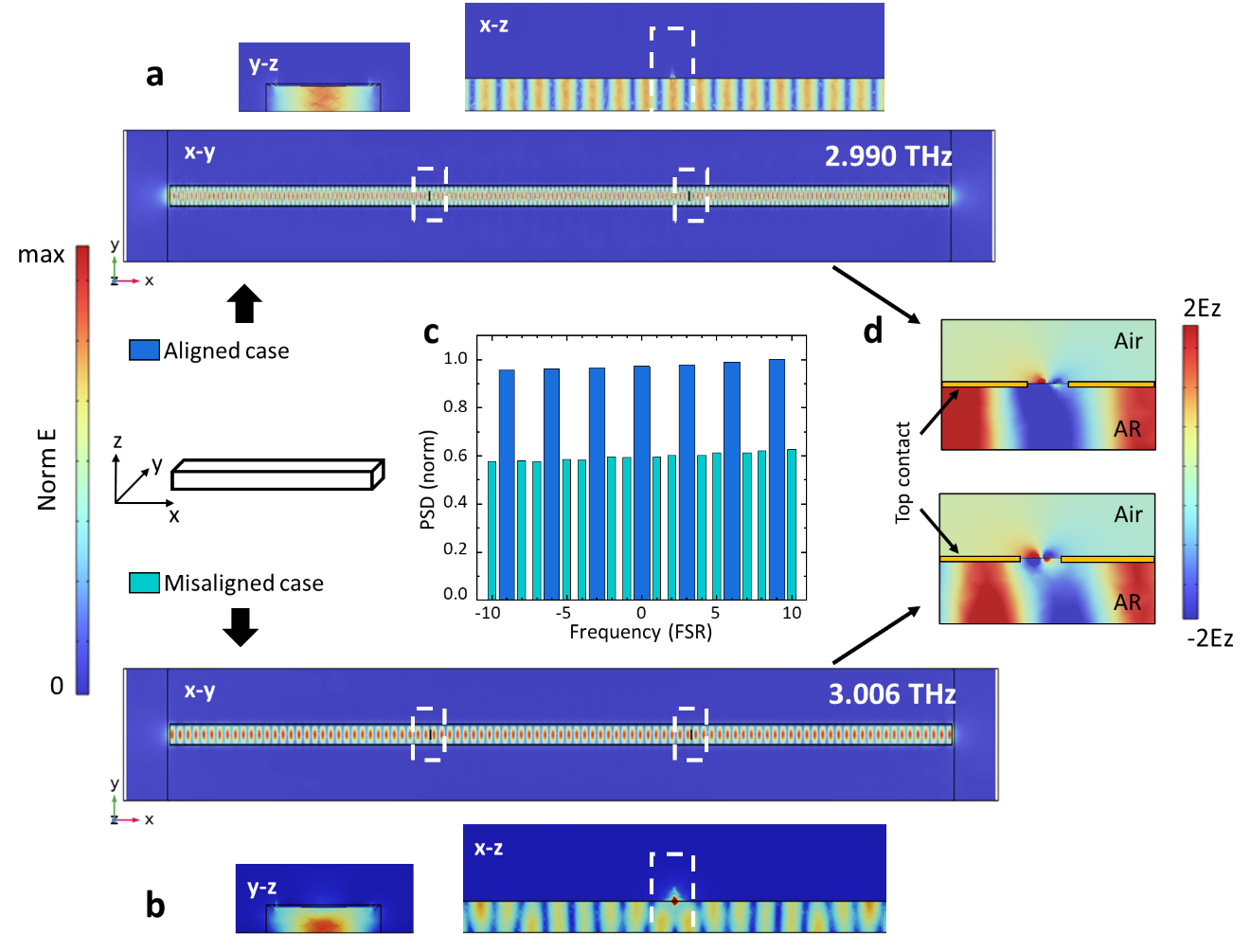
**

**Figure S1**: **a,b.** Normalized electric field amplitude simulated along the growth axis, E_z_, for the fundamental TM_00_ modes in the x-y, x-z and y-z sections along the center of the cavity. The center plot shows how the power spectral density (PSD) of the field is affected by the two graphene scatters on the top contact. This design disfavors modes that are not at the third harmonic: the field distribution for third harmonic frequencies is displayed at the top. At the bottom, it is shown the field distribution for the other frequencies where the field is perturbed more. The white dashed rectangles indicate the location of the two graphene scatters. **b.** Magnification around a scatter in the x-z plane of the E_z_ field amplified by a factor of 2.

Up to this point, the simulations of the FP cavities with graphene scatters showed consistent results with the ones obtained when Ni defects are patterned on the top laser surface, see reference [2S]. However, visible differences arise from the comparison between the electric field distribution inside the cavity and outside of the cavity, around the graphene scatters. Compared to the Ni defect cases reported in a previous work [2S], here we can notice that the magnitude of the electric field on top of the graphene scatters is smaller and less spread away from the defect surface, which is a consequence of the absorption from the graphene at terahertz frequencies.

The losses and reflectance enhancement inside the cavity, as well as the ratio between the electric field magnitude along the growth axis *z* and its total magnitude, were used to assess the effect of the graphene scatters on the HFC behaviour. The magnitude ratio represents the available energy in the cavity to excite the TM_00_ mode along the QCL. The losses in the cavity were calculated from the quality factor as $\alpha=v n_{eff}/cQ$, where $\nu$ is the frequency in Hz, $n_{eff}$ is the effective refractive index extracted from the simulations, $c$ is the velocity of light in cm/s, and $Q$ represents the quality factor. The reflectance was evaluated from the scattering parameter S11, and the electric field magnitudes was retrieved from volume integrations inside the cavity.

Figure S2 shows the ratio *E*_z_/*E*_tot_ of the reference QCL (without any opening aperture on the surface, black triangles) compared with the aligned (red squares) and misaligned (blue squares) cases of the HFC-QCL with one (Fig S2a) and two (Fig S2b) multilayer graphene scatters. In the case of the reference QCL, *E*_z_/*E*_tot_ remains almost constant at around 0.96 along the frequency range between 2.5 and 3.5 THz. On the other hand, when the peak of the *E_z_* field is aligned with the graphene defects there is almost no change in the *E*_z_/*E*_tot_ with respect to the reference QCL, less than 5%. This is true in both type of HFCs, with one and two graphene scatters. However, when the scatters are misaligned *E*_z_/*E*_tot_ decreases by about15% for the QCL with one scatters (*n* $=$ 1) and 6% for the QCL with two scatters (*n* $=$ 2).

**Figure S2.** Simulated frequency dependence of the ratio E_z_/E_tot_, between 2.5 and 3.5 THz, for the reference QCL (n $=$ 0) (back triangles) and for HFC-QCL with 1 graphene scatter, n $=$ 1 (**a**); and 2 graphene scatters, n = 2 (**b**); when the peak of the E_z_ field is aligned (red squares) and misaligned (blue squares) to the defect.

Figure S3 show the losses ratio of the QCLs simulated with one and two graphene scatters, and their respective frequency dependent losses modulation (LM). The losses ratio is calculated as the losses evaluated in the QCLs with graphene scatters divided by the losses evaluated in the reference QCL, whereas the losses modulation is calculated as the losses retrieved when the *E_z_* field is aligned divided by those extracted when it is misaligned with the scatters $ML=1-\left[ \frac{Losses (aligned}{Losses \left( misaligned \right)} \right]$. From figure S3a, it can be noticed an average increment around 5% of the losses, between 2.8 and 3.2 THz when the *E_z_* field peak is aligned with the graphene scatters and *n* $=$ 1 (red dots). Conversely, when the *E_z_* field peak is misaligned with the graphene scatters (blue dots) the losses increase by about 32% on average, in the same frequency range. These results match the ones obtained from our previous work where a defect on the top contact was considered as Ni coated (see figure S3c), however, there is a reduction of the losses ratio when multilayer graphene scatters are patterned. Similarly, figure S3b shows the losses ratio in the aligned and misaligned cases of a QCL with two graphene scatters. In this case, we observe a large increment of the losses in the aligned case while the losses ratio in the misaligned case remains almost at the same level of the same condition in QCL with one graphene scatter. Contrary to what was observed in the case of the QCL with two nickel defects (figure S3d) from reference [2S], graphene scatters produce smaller losses. Figure S3e shows the frequency dependent modulation of the losses (ML) in percentage. The blue and green curves represent the losses modulation for one graphene and two graphene scatters, respectively. As can be seen from the figure, the modulation of the losses is larger (~26%) when there is only one top graphene scatters rather than two (~5.0%).

**Figure S3** **a**. Losses ratio in the HFC QCL with 1 graphene scatter when the peak of the E_z_ field is aligned (red dots) and misaligned (blue dots) with the defect. **b.** Losses ratio in the HFC QCL with 2 graphene scatters when the peak of the E_z_ field is aligned (red dots) and misaligned (blue dots) with the defect. **c** and **d** show the losses ratio calculated when the top laser surface is patterned with one or two nickel defects aligned (orange dots) and misaligned (green dots) instead of multilayer graphene scatters. **e** Frequency dependent modulation of the losses for the HFC QCL with one single graphene scatter (blue curve) and two graphene scatters (green curve).

We iterated the same set of FEM simulations, performed on the HFCs with graphene scatters, on identical structures with open slits. Figure S4 shows the ratio E_z_/E_tot_, and PSD retrieved from the simulations performed on a QCL with one or two open scatters (not coated) in the top contact with the aim to remark the effect of the graphene coating on the scatters patterned in the top contact. The ratio E_z_/E_tot_ in the cavities with one and two open slits are shown in figure S4a and S4d, respectively. The ratio evaluated in the aligned (red squares) and misaligned (blue squares) case is compared to that obtained in the reference QCL (n = 0, black squares). As can be observed, the open slits produce an effect similar to that retrieved in presence of graphene on the field distribution inside the cavity, leading to smaller E_z_/E_tot_ ratio values when the open slits are misaligned with the E_z_ peak. However, this effect is more irregular than that obtained in the case of the graphene-coated slits.

Figures S4b and S4e show the losses ratio calculated in the QCL with one and two open slits, respectively. These results show, as in the case of the graphene-coated scatters, an increment of the losses ratio as a function of the number of scatters as well as a function of the alignment or misalignment of the scatters with respect to the E_z_ peak, but the contrast between the aligned and misaligned cases is smaller than that achieved when the scatters are graphene-coated. Furthermore, the losses ratio, in the simulated frequency range, appears more irregular when compared to the case of QCL with graphene-coated scatters, which also reflect the irregularity found in the frequency dependence of the E_z_/E_tot_ ratios.

Finally, evaluating the power spectral density (PSD) in the case of open slits (figure S4c and S4f) it is possible to observe how the contrast between the aligned and misaligned modes is significantly smaller than that achieved in both Ref. [2S] and in the present case (Figure 1c-e, in the main text), and extremely inhomogeneous, which may indicate a less efficient suppression of the misaligned modes and therefore, a not harmonic comb behavior. Moreover, from the simulation of the QCL with two open slits (Fig. S4f), the spacing, in frequency, between some of the harmonic and non-harmonic modes is not regular, oscillating between 49.4 GHz and 51.6 GHz.

**Figure S4** Simulated frequency dependence of the ratio E_z_/E_tot_, plotted between 2.5 and 3.5 THz, for the reference QCL (n = 0) (back squares) and for HFC-QCL with 1 open slit, n = 1 (**a**) and 2 open slits, n = 2 (**d**), when the peak of the E_z_ field is aligned (red squares) or misaligned (blue squares) to the defect. Losses ratio in the HFC QCL with 1 open slit (**b**) and 2 open slits (**e**), when the peak of the E_z_ field is aligned (red dots) or misaligned (blue dots) with the slit. **c, f**: Power spectral density evaluated from the simulations of the QCLs with 1 (c) and 2 (f) open slits. Yellow (c) and pink (f) colors represent the misaligned modes.

**2. Current-voltage (I-V) and light-current (L-I) characteristics**

The current density-voltage (*J*-V) and light-current density (L-*J*) characteristics of the devices presented in the main text are reported in figures. S4a-c. Compared to the reference FC QCL, the HFCs show a 2-9% increase of the threshold current density (*J*_th_), consistent with the presence of an increased number of multilayer graphene scatters on the top surface, with a corresponding 8-42% increase of the slope efficiency, and a variation of the peak optical power about 6% (Table S1). This proves that the multilayer graphene scatters redistribute the power of the suppressed modes into the harmonic modes, only partially impacting the overall device performance.

**Figure S5.** Current density-voltage (J-V) and light-current density (L-J) characteristics of the reference FC (a), the HFC with one graphene defect, corresponding to the second harmonic QCL (b), and the HFC with two graphene defects, corresponding to the third harmonic QCL (c).

|  | **J_th_ (A/cm^2^)** | **Slope Efficiency** | **Maximum WP efficiency (%)** | **Peak optical power (mW)** |
| --- | --- | --- | --- | --- |
| ***n* = 0** | 340.38 $\pm$ 7.6 | 31.26 $\pm$ 4.0 | 0.079 $\pm$ 2x10^-3^ | 3.8 $\pm$ 0.08 |
| ***n* = 1** | 348.63 $\pm$ 7.6 | 26.58 $\pm$ 4.0 | 0.067 $\pm$ 2x10^-3^ | 4.2 $\pm$ 0.08 |
| ***n* = 2** | 369.52 $\pm$ 7.6 | 22.02 $\pm$ 4.3 | 0.04 $\pm$ 2x10^-3^ | 3.6 $\pm$ 0.08 |
| **Mean** | 352.84 $\pm$ 4.4 | 26.61 $\pm$ 2.13 | 0.062 $\pm$ 1.2x10^-3^ | 3.86 $\pm$ 0.08 |
| **Standard Deviation** | 12.26 | 3.76 | 0.008 | 0.30 |

**Table S1.** Figures of merit of the devices described in the main text

**3. Harmonic frequency comb behavior**

Figure S6 shows the comparison between two HFC QCLs fabricated with one graphene scatter and one nickel defect, on the top contact with identical dimensions, 2.5 mm length and 70 um width. Figs S5a,c show the spectrum and interferogram corresponding to the HFC with 1 graphene scatter, respectively, whereas Figs S5b,d show the corresponding to an HFC with one nickel defect. The combination of the FTIR spectra and intermode beatnote map analysis, show that both devices behave as 2^nd^ harmonic frequency combs, with emission centered around 3 THz, however, the interferograms show a fully different behavior under the same harmonic response. The retrieved interferogram in the case of the sample with one graphene scatter shows a pattern composed by two lobes of different intensities which alternate periodically and symmetrically, that is regularly found in the case of emission of pulses. On the other hand, the interferogram from the device with one nickel defect exhibit a rather different pattern, symmetrically, but with no evidence of any pulsing-like pattern.

**Figure S6:** Comparison between two HFCs with one graphene scatter and one nickel defect. **a** and **b** show the spectra from the HFC with one graphene scatter and one nickel defect, respectively. **c** and **d** show the interferograms corresponding to the spectra of the same color.

**References**

[1S] Angeles, L. *Optical Properties of Graphene from the THz to the Visible Spectral Region*. University of California. 2012.

[2S] Riccardi, E., Alejandro, M., Guerrero, J., Pistore, V., Seitner, L., Jirauschek, C., Li, L., Davies, A. G., Linfield, E. H., & Vitiello, M. S. (2023). “Sculpting harmonic comb states in terahertz quantum cascade lasers by controlled engineering.” <https://arxiv.org/abs/2311.03123v1>
